# ORGANIZATIONAL PRACTICES FOR THE AGING WORKFORCE: A CROSS-CULTURAL VALIDATION OF THE LATER-LIFE WORKPLACE INDEX

**DOI:** 10.1093/geroni/igac059.2486

**Published:** 2022-12-20

**Authors:** Juergen Deller, Julia Finsel, Anne Wöhrmann, Max Wilckens, Xiuzhu Gu, Eduardo Oliveira

**Affiliations:** Leuphana University of Lueneburg, Lueneburg, Niedersachsen, Germany; Leuphana University Lueneburg, Lüneburg, Niedersachsen, Germany; BAuA, Dortmund, Nordrhein-Westfalen, Germany; Leuphana University Lueneburg, Lüneburg, Niedersachsen, Germany; Tokyo Institute of Technology, Tokyo, Tokyo, Japan; University of Porto, Porto, Porto, Portugal

## Abstract

Successful employment of experienced employees becomes more important for both, individuals and organizations. To identify organizational practices that foster the motivation, health, and performance of experienced employees in particular, a holistic assessment of relevant organizational factors is needed. The Later Life Workplace Index (LLWI) provides such a measure for organizational practices for older employees by differentiating nine domains, namely organizational climate, leadership, work design, health management, individual development, knowledge management, transition to retirement, continued employment after retirement, and health and retirement coverage. So far, a German-language and an English-language version of the LLWI have been validated in Germany and the U.S in a multi-study procedure. The psychometric properties and measurement invariance of the English-language version of the LLWI will be presented. Preliminary findings from Japan and Portugal show promising results regarding reliability and validity of the LLWI in the respective country. The findings suggest that the multidimensional measurement model developed in Germany and the U.S. could be applicable to other regulatory and cultural contexts as well. A focus group consisting of the original authors of the LLWI and international scholars, whose research expertise lies in the field of employment and older employees, is currently developing a short version of the LLWI. We aim to provide researchers and practitioners from different countries with a validated measurement to holistically assess organizational practices. Researchers can utilize the LLWI to gain a comprehensive understanding of organizational influences on later life work, while practitioners are able to assess their organizational readiness for an aging workforce.

